# Neurosensory Disturbances Related to the Inferior Alveolar Nerve Amongst Patients With Mandibular Medication-Related Osteonecrosis of the Jaw (MRONJ): A Clinical and Radiological Overview

**DOI:** 10.1016/j.identj.2026.109643

**Published:** 2026-05-27

**Authors:** Yui Yin Ko, Wei-fa Yang, Yiu Yan Leung

**Affiliations:** Division of Oral and Maxillofacial Surgery, The University of Hong Kong, Sai Ying Pun, Hong Kong SAR, China

**Keywords:** MRONJ, Neurosensory disturbances, Inferior alveolar nerve, Medication-related osteonecrosis of the jaw

## Abstract

**Introduction and aims:**

The aims of this study were to evaluate the clinical and radiological characteristics of patients diagnosed with mandibular medication-related osteonecrosis of the jaw (MRONJ) and to examine its associations with neurosensory disturbances (NSD) related to the inferior alveolar nerve.

**Methods:**

A retrospective review was conducted of all patients diagnosed with MRONJ who had undergone cone-beam computed tomography (CBCT) or computed tomography (CT) imaging in the Department of Oral and Maxillofacial Surgery at the Prince Philip Dental Hospital between January 2013 and August 2024. Clinical characteristics and the radiological manifestations of the mandibular canal were extracted. Statistical analyses were carried out with the significance level set at 5%.

**Results:**

A total of 61 patients with 65 MRONJ lesions were included, of which 21 lesions (32.3%) exhibited neurosensory disturbances. The most common radiological manifestation of the MC was osteosclerotic (60%) and osteolytic changes (60%), followed by sequestration (46.2%). Multivariate logistic regression analysis identified active infection (adjusted OR 4.91, 95% CI: 1.14-21.2, *p* = .033) and sequestrum impingement (adjusted OR: 6.17, 95% CI: 1.76-21.7, *p* = .005) as significant associations of NSD. Four out of 15 patients with active infection and preintervention NSD presented with complete resolution of NSD following the elimination of the infection.

**Conclusion:**

Approximately one-third of mandibular MRONJ lesions presented with NSD. Sequestration impinging on the MC and active infection are associated with the presence of NSD.

**Clinical relevance:**

The removal of sequestrum and treatment of infection may lead to an improvement in pre-intervention NSD; therefore, the inferior alveolar nerve should not be sacrificed routinely during the operation.

## Introduction

MRONJ, characterized by persistent exposed bone in the jaw, is a debilitating complication of antiresorptive or anti-angiogenic medications. While the majority of patients are treated with antiresorptive medications for osteoporosis,^1^^,^^2^ these drugs are also implicated in the management of malignancy-related conditions, including malignancy-associated hypercalcemia and the prevention of skeletal-related events associated with bone metastases in solid tumours and multiple myelomas.^3^, ^4^, ^5^, ^6^, ^7^

Numb chin syndrome (NCS), also known as Vincent’s symptom, refers to neurosensory disturbances (NSD) along the distribution of the inferior alveolar nerve (IAN) or mental nerve. The most common causes of NCS are iatrogenic damage by dentoalveolar procedures, maxillofacial trauma, or odontogenic infections.^8^ While NCS can be the first presenting symptom of MRONJ, it can also be an early manifestation of multiple sclerosis or an indicator of systemic malignancy or bony metastasis.^8^, ^9^, ^10^ NCS has been reported as the first presenting symptom in multiple malignancies, including lymphoma, multiple myeloma, and metastatic renal, lung, prostate, and breast cancers.^8^^,^^9^^,^^11^, ^12^, ^13^, ^14^

NSD as a presenting symptom of MRONJ was first reported by Otto et al., in which 4 mandibular MRONJ cases were diagnosed at early stages.^15^ Fortunato et al. further evaluated the clinical presentations of 29 patients with mental neuropathy but no clear odontogenic causes. In this study, NCS presented as the first symptom of systemic malignancy in 11 patients and as the first symptom of prodromal MRONJ in 13 patients.^16^ Lu et al. reviewed 16 patients with NCS, of which only 19% were caused by MRONJ, while 75% were due to malignancy-associated mandibular or intracranial invasion.^17^ In the context of metastatic disease, NCS is a poor prognostic indicator, with only 15% of patients surviving more than 9 months.^11^ Considering that patients with solid tumours are often on MRONJ-associated medications, it is crucial for clinicians to differentiate between metastatic jaw lesions and MRONJ to enable prompt intervention and appropriate referrals.

The identification of clinical and radiological characteristics associated with NSD in mandibular MRONJ may benefit clinicians in differential diagnosis. To date, this has never been explored in depth in medical literature. Only a retrospective study by Duygu Goller-Bulut et al. evaluated the radiological presentation of neurovascular bundles in MRONJ lesions, finding that they had narrower diametric measurements in canals and foramina.^18^ However, the association of such radiological presentations with NSD was not further investigated. Hence, this study aimed to evaluate the role of NSD related to the IAN in mandibular MRONJ lesions and their associated clinical and radiological characteristics.

## Materials and methods

### Study population

The operating lists and clinic booking records of the Department of Oral and Maxillofacial Surgery at the Faculty of Dentistry, The University of Hong Kong, were screened to identify eligible patients between 1st March 2013 and 31st March 2025. The inclusion criteria were patients who were diagnosed with Stage 0, 1, 2, 3 mandibular MRONJ in accordance with the diagnostic criteria outlined in the AAOMS position paper of 2022 and had undergone cone-beam computed tomography (CBCT) or computed tomography (CT) imaging. The diagnostic criteria of MRONJ outlined in the AAOMS position paper of 2022 are as follows: ^1^

1. Current or previous treatment with antiresorptive therapy alone or in combination with immune modulators or antiangiogenic medications.
2. Exposed bone or bone that can be probed through an intraoral or extraoral fistula(e) in the maxillofacial region that has persisted for more than eight weeks.
3. No history of radiation therapy to the jaws or metastatic disease to the jaws.

The exclusion criteria were patients who had incomplete clinical information regarding the status of NSD during the initial consultation and ineligible or unavailable radiologic images. Patients who received imaging following surgical intervention and those with imaging modalities with unacceptable diagnostic standards (severe motion or metal artifacts) were also excluded. Ethical approval was sought from the Institutional Review Board of the University of Hong Kong/Hospital Authority Hong Kong West Cluster (IRB reference number: UW 25-213).

### Data collection

#### Clinical characteristics

Clinical variables were collected by a retrospective chart review of clinical records at the Prince Phillip Dental Hospital. Demographic data collected included age at diagnosis, gender, systematic comorbidities, history of smoking and alcohol use, history of MRONJ-associated medications (including indications, type of medication, route of administration, length of administration), chronic corticosteroid or immunosuppressive therapy. Clinical data including the location of MRONJ lesion, the disease staging of MRONJ in accordance with the AAOMS position papers and the type of intervention (surgical or non-surgical) delivered.^1^ Surgical interventions were defined as sequestrectomies and marginal or segmental jaw resections. The status of NSD during the initial consultation and one month following the delivery of intervention were also recorded. The intra-operative events related to the integrity of IAN during surgical intervention were also recorded. The location of MRONJ lesion was recorded depending on the laterality.

## Radiological characteristics

### Image acquisition

CBCT, or Spiral CT was acquired for each patient following initial consultation. CBCT examination was performed with Planmeca ProMax 3D Classic (Planmeca Oy) and Spiral CT examination was performed with Revolution ACT (GE Healthcare). The field of view ranged from 8×5 cm to 20×10cm and was determined according to the patient’s diagnostic and therapeutic indications, with voxel size 400 micrometers and with an operative range at 90kV, 5.6-8mA, scan time 4.569-13.511s. Data in the format of digital imaging and communication in medicine (DICOM) was imported to RadiAnt DICOM Viewer 2025.1 (64-bit) (Medixant) for imaging reformatting.

### Radiological assessment

All radiological assessments were carried out on a DELL P2014H LED monitor with a resolution of 1600 × 900 pixels (DELL, Texas, US). Image brightness, contrast, and magnification settings were adjusted at the observer’s discretion using the device-inherent software (RadiAnt DICOM Viewer 2025.1 [64-bit] [Medixant]). In spiral CT images, the threshold was adjusted to a bony window to improve visualization of osseous changes. When multiple radiological examinations were conducted, the imaging taken within 1 month of its corresponding clinical examination was adopted to allow clinical correlation.

**Fig. 1:**
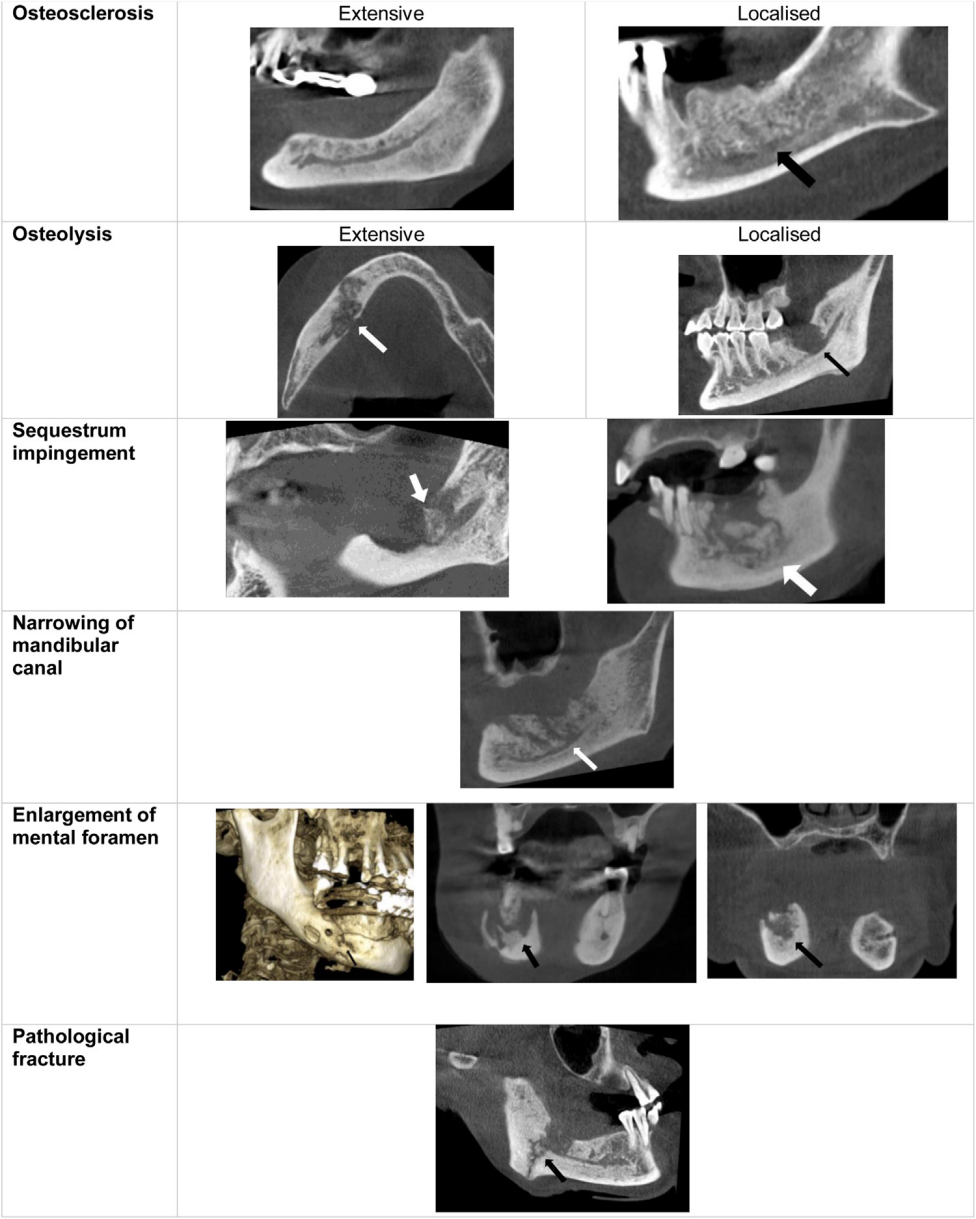
Examples of radiological changes in the MC in MRONJ lesions on CBCT images.

The imaging assessment was performed for each MRONJ lesion. The radiological evaluation included the following osseous changes of the mandibular canal (MC) (Fig. 1):

1. Osteosclerosis. This refers to radiopaque regions. The lesions were classified as “localised” if they extended to 1 cm and “extensive” if they exceeded 1 cm.
2. Osteolysis. This refers to radiolucent areas involving the cortical/and or trabecular bone. The lesions are classified as following
  • “Extensive”: Erosion of all walls of the mandibular canal
  • “Localised”: Erosion of the superior wall of the mandibular canal
  • “Absent”
3. Sequestration refers to the formation of a bony island surrounding by an osteolytic halo. The presence of sequestrum impinging the MC was recorded.
4. Narrowing of MC
5. Enlargement of mental foramen
6. Pathological fractures

### Outcome measures

The primary outcome measure was to evaluate the radiological changes to the MC amongst patients with mandibular MRONJ. The secondary outcome was to evaluate the status of NSD prior to and following the delivery of interventions and its association with clinical and radiological characteristics.

### Statistical analysis

All data were analysed descriptively. Intraobserver reliability was evaluated by repeating the radiological assessment for 20 cases chosen at random by the same investigator, and intraclass correlation coefficients were derived. Intraobserver reliability was considered poor when the values are less than 0.5, moderate when 0.5 to 0.75, good when 0.75 to 0.90, and excellent when greater than 0.90.^19^

The Chi-square test was used to detect associations between the presence of clinical and radiological features and the presence of NSD. The Fisher’s exact test was used when more than 20% of the cells had expected frequencies less than 5. A multivariate logistic regression analysis was developed based on clinical and radiological variables which were statistically significant in univariate analysis at the *p*-value <.05 level. Statistical analysis was performed using IBM Statistical Package for Social Sciences version 29.0.2.0 software (IBM Corp.), with a 5% significance level applied.

## Results

The study sample initially consisted of 71 patients and 76 mandibular MRONJ lesions. Of which, 10 patients were excluded – 8 patients did not have available or eligible imaging, 1 patient did not undergo clinical examination at the Department and 1 patient underwent imaging following surgical intervention. Therefore, the final study sample was composed of 65 mandibular lesions in 61 patients. The majority of patients (59/61, 96.7%) underwent CBCT radiological examination.

The majority of patients were female (55, 90.2%), with a mean age of 76.0 years old (Range: 40.0-96.0, S.D. 11.6). The frequency of NSD in this study was 21 lesions (32.3%). Table 1 outlined the descriptive demographics of the study cohort. Amongst the 7 clinical variables investigated, only the presence of active infection was shown to be associated with NSD. (*p* = .021) (Table 2)

**Table 1:** Demographics and clinical characteristics of the study cohort (N = 61).

	Number (%)
Gender	
Male	6 (9.8%)
Female	55 (90.2%)
Age	
Range	40.0 - 98.6
Mean (S.D.)	76.0 (11.6)
Primary disease	
Benign (Osteoporosis)	46 (75.4)
Malignancy	15 (24.6)
Origins of malignant disease (N=15)	
Metastatic Breast Cancer	9 (60)
Metastatic Lung Cancer	2 (13.3)
Multiple Myeloma	4 (26.6)
Concurrent corticosteroid use	
Yes	8 (13.1)
No	53 (86.9)
Duration of medication	
Less than 5 years	37 (60.7)
5 or more years	24 (39.3)
Route of administration	
Oral Bisphosphonates	39 (63.9)
Intravenous bisphosphonates	12 (19.7)
Subcutaneous denosumab	10 (16.4)

**Table 2:** Associations between clinical variables and presence of NSD (n = 65).

Clinical variable		Total (N, %)	NSD (N, %)		*p*-value
			Present	Absent	
Gender	Male	8(12.3)	1 (1.5)	7 (10.8)	.259*
	Female	57(87.7)	20 (30.8)	37 (56.9)	
Route of administration	Oral Bisphosphonates	41 (63.1)	13 (20.0)	28 (43.1)	.117
	Subcutaneous denosumab	10 (15.4)	1 (1.5)	9 (13.8)	
	Intravenous Bisphosphonates	14 (21.5)	7 (10.8)	7 (10.8)	
Duration of medications	Less than 5 years	40 (61.5)	14 (21.5)	26 (40.0)	.557
	5 or more years	25 (38.5)	7(10.8)	18 (27.7)	
Primary disease	Benign	48 (73.8)	15 (23.1)	33 (50.8)	.759
	Malignancy	17 (26.2)	6 (9.2)	11 (16.9)	
Clinical staging	0	2 (3.10)	1 (1.54)	1(1.54)	.322*
	1	15 (23.1)	3 (4.6)	12 (18.5)	
	2	36 (55.4)	11 (16.9)	25 (38.5)	
	3	12 (18.5)	6 (9.2)	6 (9.2)	
Active infection	Present	43 (66.2)	18 (27.7)	25 (38.5)	.021
	Absent	22 (33.8)	3 (4.6)	19 (29.2)	
Concurrent corticosteroid use	Present	9 (13.8)	3 (4.6)	6 (9.2)	1.000*
	Absent	56 (86.2)	18 (27.7)	38 (58.5)	

⁎ Fisher’s exact test

Regarding radiological characteristics, the most common presentation was osteosclerotic (39, 60%) and osteolytic changes (39, 60%), followed by sequestration (30, 46.2%). Lesions with sequestrum impinging into MC and mandibular fractures involving the MC were more likely to be associated with NSD than those without (Table 3).

**Table 3:** Associations between radiological variables and presence of NSD (n = 65)

Radiological manifestations of the mandibular canal		Total (n, %)	NSD (n, %)		*p*-value
			Present	Absent	
Osteosclerosis	Extensive	14 (21.5)	5 (7.7)	9 (13.8)	.95
	Localised	25 (38.5)	8 (12.3)	17 (26.2)	
	Absent	26 (40)	8 (12.3)	18 (27.7)	
Osteolysis	Extensive	13 (20)	7 (10.8)	6 (9.2)	**.037**
	Localised	26 (40)	10 (15.4)	16 (24.6)	
	Absent	26 (40)	4 (6.2)	22 (33.8)	
Narrowing of MC	Present	2 (3.1)	1 (1.5)	1 (1.5)	.545*
	Absent	63 (96.9)	20 (30.8)	43 (66.2)	
Sequestration	Impingement	18 (27.7)	11 (16.9)	7 (10.8)	**.008**
	Absent	35 (72.3)	10 (29.2)	28 (43.1)	
Mandibular fracture	Present	3(4.6)	3 (4.6)	0 (0)	.03*
	Absent	62 (95.4)	18 (27.7)	44 (67.7)	
Destruction of mental foramen	Present	10 (15.4)	4 (6.2)	6 (9.2)	.715*
	Absent	55 (84.6)	17 (26.2)	38(58.5)	

⁎ Fisher’s exact test

Univariate logistic regression analysis identified the radiographic features of sequestrum impingement (unadjusted OR 5.81, 95% CI 1.79-18.9, *p* = .003) and presence of active infection (unadjusted OR: 4.56, 95% CI: 1.17-17.8, *p* = .029) as being statistically associations of NSD in MRONJ lesions (Table 4). Multivariate logistic regression analysis identified active infection (adjusted OR: 4.91, 95% CI: 1.14-21.2, *p* = .033) and sequestrum impingement (adjusted OR: 6.17, 95% CI: 1.76-21.7, *p* = .005) as significant associations of NSD.

**Table 4:** Univariate logistic regression analysis to identify variables associated with NSD.

	Odds ratio (OR)	95% Confidence interval (CI)	*p*-value
*Gender*			
Male (ref)	1		
Female	3.78	0.434-33.0	.228
*Primary disease*			
Benign (ref)	1		
Malignancy	1.20	0.374-3.86	.759
*Concurrent corticosteroid use*			
No (ref)	1		
Yes	1.06	0.237-4.71	.943
*Duration of medication*			
Less than 5 years (ref)	1		
5 or more years	0.722	0.243-2.14	.558
*Route of administration*			
Oral bisphosphonates/Subcutaneous (ref)	1		
Intravenous bisphosphonates	2.64	0.784-8.91	.117
*Clinical staging*			
Stage 0 /1 (ref)	1		
Stage 2	1.43	0.38-5.39	.597
Stage 3	3.25	0.661-16.0	.147
*Presence of active infection*			
None (ref)	1		
Present	**4.56**	**1.17-17.8**	**.029**
*Radiographic sign*			
None(ref)	1		
Sequestrum impingement	**5.81**	**1.79-18.9**	**.003**
Extensive osteolysis	3.17	0.91-11.1	.071
Narrowing of MC	2.15	0.128-36.1	.595
Mental foramen destruction	1.49	0.372-5.97	.573
Mandibular fracture	3940000000	0-Inf.	.999
Extensive osteosclerosis	1.2	0.351-4.21	.759

### Post-operative recovery

Post-intervention neurosensory status was assessed for 46 lesions. Among these lesions, 36 underwent surgical intervention. Of the lesions that did not present with pre-operative NSD, 79.2% (n = 19) did not experience post-operative NSD. In contrast, 91.7% (n = 11) of lesions that presented with pre-operative NSD presented with NSD post-operatively. Of the 11 patients with post-operative NSD, 5 had transection of the IAN intra-operatively. Out of the 15 patients with active infection and pre-intervention NSD, 4 presented with resolution of NSD following the elimination of the infection.

The intraclass coefficient for absolute agreement was 0.90 for the assessment of osteosclerosis, 0.95 for the assessment of sequestration and osteolysis and 1.00 for the assessment of narrowing of MC, mental foramen destruction and mandibular fracture. Therefore, intraobserver reliability was excellent.

## Discussion

Given the malignancy background in MRONJ patients, it’s crucial to differentiate MRONJ from possible malignancy in the clinical presence of NCS. The results of this study aim to assist clinicians by highlighting the radiological manifestations related to the MC in MRONJ lesions. The key findings of our study indicated that the presence of active infection and sequestrum involvement are associated with NSD among patients diagnosed with mandibular MRONJ.

The prevalence of NSD among MRONJ patients is rarely reported, and its mechanism remains unexplained. NSD as a symptom of MRONJ was first documented by Otto et al. in 2009.^15^ In this case series of 4 patients, 3 were diagnosed at early stages, and improvement in neurosensory function was noted following surgical interventions. Retrospective studies reported clinical manifestations of paraesthesia to range from 8.2% to 25%.^20^, ^21^, ^22^ Our current study presents a prevalence of 20% of NSD, which is consistent with previous studies.

Osteosclerosis was found to be the most observed manifestation related to the MC in our study. Shin et al. found that 3 out of 20 MRONJ cases exhibited sclerosis encroaching on the MC.^23^ Similarly, another study by Phal et al. found the incidence of sclerotic changes around the MC to be around 30%.^24^ This incidence is much lower compared to our cohort, which may result from inconsistencies in the radiological imaging modalities used in such studies, including 2-dimensional panoramic images and nuclear imaging. Three-dimensional CT or CBCT is more accurate than 2-dimensional imaging in delineating osseous lesion extent and is considered the gold standard for evaluating MRONJ.^25^^,^^26^

Jaw metastatic lesions are known to mimic MRONJ lesions, making differentiation difficult in a clinical setting. In our study, the presence of sequestrum impingement was statistically correlated with NSD, which may serve as a potential radiological indicator to help differentiate between MRONJ and metastatic jaw lesions in patients with NCS. Sequestrum formation is a characteristic radiological finding in MRONJ and is less commonly found in metastatic jaw lesions. Yfanti et al. compared the CBCT images of both disease entities and noted sequestration in 69.4% of MRONJ lesions as opposed to 36.4% in metastatic jaw lesions.^27^ Another study by Gaêta-Araujo et al. further verified this finding, showing sequestration in 56.5% of MRONJ lesions compared to 15.8% in jaw metastatic lesions.^28^

In our study, the presence of active infection was associated with NSD. This has been hypothesized to result from 2 mechanisms: the first being infection-related, arising from intrabony purulent exudate or ischemia associated with inflammatory edema causing mechanical pressure on the nerve. The second mechanism involves chemical damage to the nerve due to toxic metabolites or inflammatory products released from tissue damage.^29^^,^^30^ To date, there have been no longitudinal studies conducted to evaluate neurosensory recovery following infection-related paraesthesia. Nevertheless, multiple case reports and case series have demonstrated complete resolution of disturbances following the elimination of the infective source.^29^, ^30^, ^31^, ^32^ This is further supported by our findings, in which 4 out of 15 patients gained full neurosensory recovery following intervention.

The prevalence of NSD following surgery in our study is 39.1%. While the prevalence of NSD after sequestrectomy has not been previously reported, it appears comparable to the temporary NSD rates following bilateral sagittal split osteotomies.^33^^,^^34^ The relatively high incidence of temporary NSD may be due to challenges in lesion demarcation during sequestrectomy. At our center, necrotic bone removal is routinely performed using conventional rotary osteotomy until bleeding margins are reached.^35^, ^36^, ^37^ Additionally, our data only recorded NSD status 1 month postintervention, while complete recovery from IAN damage can take up to 24 months, typically showing a bimodal recovery pattern after 6 and 9 months.^38^ Thus, in cases where the neurovascular bundle is preserved, the rate of permanent NSD after sequestrectomy may be lower.

In our cohort, the IAN was sacrificed in 5 cases due to lesion involvement. However, previous studies suggest prioritizing IAN preservation, especially in benign cases, as it can maintain function even with extensive bony destruction.^39^ IAN injury can lead to functional deficits such as drooling, poor articulation, and psychological impacts on daily activities and social interactions.^40^^,^ ^41^ Patients undergoing IAN-preserving resection for benign pathologies report a better quality of life compared to those whose nerves were sacrificed.^39^

Previous studies reported various methods for protecting and preserving the IAN during surgeries. One method involves the application of piezosurgery, which has demonstrated superior postoperative sensory recovery for the removal of sequestrum in close proximity to the IAN in MRONJ lesions compared to conventional handpieces.^42^ In the latest AAOMS position papers, more aggressive surgical approaches have been recommended for patients who are deemed medically fit to undergo surgery under general anaesthesia. Marginal resection is recommended for localised diseases above the neurovascular canal and segmental resection for diseases extending beyond it.^1^ With the emerging technologies of Computer-Aided Design and Computer-Aided Manufacturing (CAD-CAM) and 3D printing, the use of patient-specific templates has been advocated to aid in IAN preservation. Huang et al. reported the use of templates to guide osteotomy cuts to precisely avoid the IAN during marginal mandibulectomy,^43^ while Tereshchuk et al. adopted templates to aid demarcating the nerve position to enable nerve lateralization prior to performing segmental resection.^39^ Chen et al. advanced the field further by adopting a dynamic navigation system to provide real-time guidance on the position of the inferior dental nerve intraoperatively by registering the jaw to preoperative CT images.^42^

The present study is not without its limitations. Firstly, it is a retrospective study; thus, certain clinical information was unavailable as it is not routinely collected or recorded, resulting in selection bias. This includes standardized patient-reported and clinician-reported neurosensory testing, as well as comparisons of longitudinal NSD status after interventions. Secondly, given the rare nature of MRONJ, the patient cohort is relatively small, which may limit statistical significance. Thirdly, there is a lack of a control group for comparison; patients with metastatic mandibular lesions could serve as a good control to draw more definitive conclusions regarding the differences in radiological manifestations of the MC. Future prospective multicentre studies with a larger sample size and standardized longitudinal neurosensory testing protocols could be valuable in validating our findings.

## Conclusion

This study highlighted that the presence of active infection and sequestrum involvement could be associated with NSD in mandibular MRONJ. In contrast to previous findings, NSD is more commonly observed in lesions of higher clinical staging. For lesions with IAN involvement, IAN-preserving sequestrectomies or resection surgeries should be performed, as the removal of sequestrum or active infection is likely to result in improvement or even resolution of NSD.

## Conflicts of interest

The author is an Editorial Board Member for this journal and was not involved in the editorial review or the decision to publish this article. The authors declare that they have no other competing financial interests or personal relationships that could have appeared to influence the work reported in this paper.
